# *N*′-(2,4-Di­nitro­phen­yl)acetohydrazide monohydrate

**DOI:** 10.1107/S1600536813018916

**Published:** 2013-07-13

**Authors:** Manel Essid, Houda Marouani, Salem S. Al-Deyab, Mohamed Rzaigui

**Affiliations:** a Laboratoire de Chimie des Matériaux, Faculté des Sciences de Bizerte, 7021 Zarzouna Bizerte, Tunisia; b Chemistry Department, Faculty of Science, King Saud University, PO Box 2455, Riyadh 11451, Saudi Arabia

## Abstract

In the crystal structure of the title compound, C_8_H_8_N_4_O_5_·H_2_O, the organic and lattice water mol­ecules are linked together *via* N—H⋯O and O—H⋯O hydrogen bonds. A C—H⋯O inter­action is also observed between the organic mol­ecules. These hydrogen bonds and inter­actions lead to the formation of a three-dimensional network. An intra­molecular N—H⋯O hydrogen bond also occurs. The dihedral angle between the acetyl group and the almost planar hydrazide moiety [maximum deviation from the least-squares plane is 0.209 (2) Å for one of the nitro O atoms] is 88.5 (3)°.

## Related literature

For background to the biological activity of hydrazines and hydrazones, see: Zahid & Sherazi (1997 ▶); Monfared *et al.* (2007 ▶). For a related crystal structure, see: Okabe *et al.* (1993 ▶). For hydrogen-bond graph-set motifs, see: Bernstein *et al.* (1995 ▶).
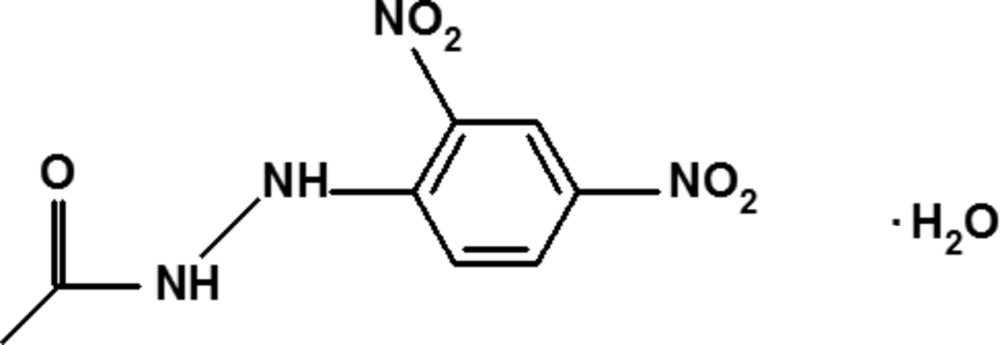


## Experimental

### 

#### Crystal data

C_8_H_8_N_4_O_5_·H_2_O
*M* *_r_* = 258.20
Monoclinic, 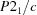

*a* = 7.702 (2) Å
*b* = 7.057 (3) Å
*c* = 21.550 (4) Å
β = 109.044 (19)°
*V* = 1107.3 (6) Å^3^
*Z* = 4
Ag *K*α radiation
λ = 0.56083 Å
μ = 0.08 mm^−1^
*T* = 293 K
0.5 × 0.4 × 0.3 mm

#### Data collection

Enraf–Nonius CAD-4 diffractometer
7469 measured reflections
5411 independent reflections
2771 reflections with *I* > 2σ(*I*)
*R*_int_ = 0.064
2 standard reflections every 120 min intensity decay: 1%

#### Refinement

*R*[*F* ^2^ > 2σ(*F* ^2^)] = 0.071
*wR*(*F* ^2^) = 0.195
*S* = 0.98
5411 reflections
181 parameters
3 restraints
H atoms treated by a mixture of independent and constrained refinement
Δρ_max_ = 0.25 e Å^−3^
Δρ_min_ = −0.23 e Å^−3^

### 

Data collection: *CAD-4 EXPRESS* (Enraf–Nonius, 1994 ▶); cell refinement: *CAD-4 EXPRESS*; data reduction: *XCAD4* (Harms & Wocadlo, 1995 ▶); program(s) used to solve structure: *SHELXS86* (Sheldrick, 2008 ▶); program(s) used to refine structure: *SHELXL97* (Sheldrick, 2008 ▶); molecular graphics: *ORTEP-3 for Windows* (Farrugia, 2012 ▶) and *DIAMOND* (Brandenburg & Putz, 2005 ▶); software used to prepare material for publication: *WinGX* (Farrugia, 2012 ▶).

## Supplementary Material

Crystal structure: contains datablock(s) I, global. DOI: 10.1107/S1600536813018916/pv2637sup1.cif

Structure factors: contains datablock(s) I. DOI: 10.1107/S1600536813018916/pv2637Isup2.hkl

Supplementary material file. DOI: 10.1107/S1600536813018916/pv2637Isup3.cml

Additional supplementary materials:  crystallographic information; 3D view; checkCIF report

## Figures and Tables

**Table 1:** Hydrogen-bond geometry (Å, °)

*D*—H⋯*A*	*D*—H	H⋯*A*	*D*⋯*A*	*D*—H⋯*A*
O*W*—H1*W*⋯O5	0.86 (1)	1.91 (1)	2.759 (3)	175 (3)
O*W*—H2*W*⋯O4^i^	0.84 (1)	2.05 (1)	2.868 (3)	165 (3)
N1—H1*N*⋯O*W* ^ii^	0.88 (3)	2.04 (3)	2.883 (3)	160 (2)
N2—H2*N*⋯O1	0.90 (3)	1.96 (3)	2.607 (2)	128 (2)
N2—H2*N*⋯O*W* ^iii^	0.90 (3)	2.15 (3)	2.910 (3)	142 (2)
C6—H6⋯O5^iv^	0.93	2.40	3.310 (3)	165

Symmetry codes: (i) 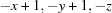; (ii) 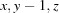; (iii) 
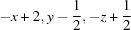
; (iv) 
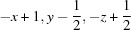
.

**Fig. 1.**
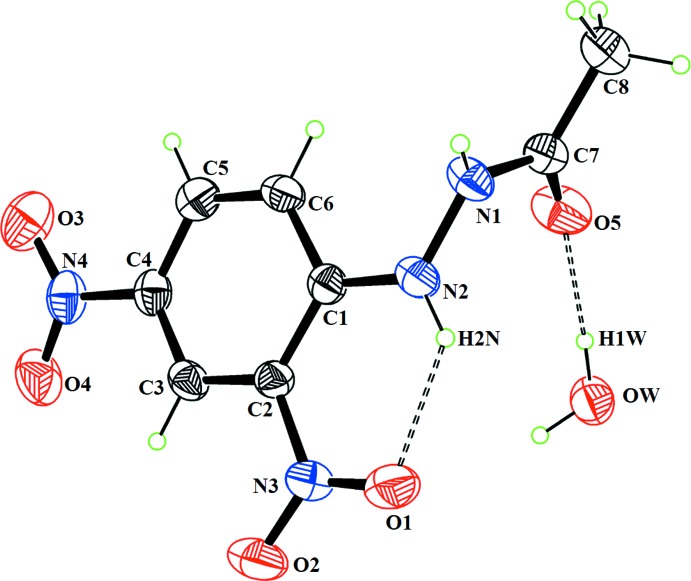
An ORTEP view of the title compound with the atom-labelling scheme. Displacement ellipsoids are drawn at the 30% probability level. H atoms are represented as small spheres of arbitrary radii. Hydrogen bonds are represented as dashed lines.

**Fig. 2.**
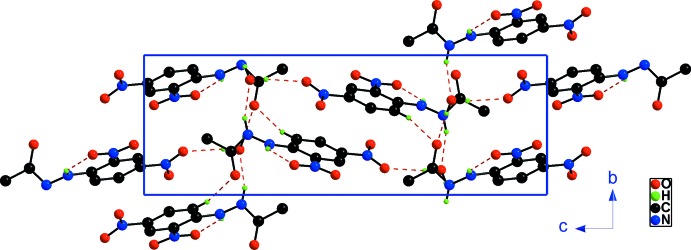
Unit cell packing of the title compound projected along the a axis. The H-atoms not involved in H-bonding are omitted.

## supplementary crystallographic information

### Comment

Hydrazine and its derivatives have been intensively investigated due to their attractive pharmacological applications (Zahid & Sherazi, 1997). In addition, hydrazones exhibit physiological activities in the treatment of several diseases such as tuberculosis (Monfared *et al.*, 2007). We report here the synthesis and the crystal structure of the title compound.

The bond distances and angles in the title compound (Fig. 1) agree very well with the corresponding bond distances and angles reported in a closely related compound (Okabe, *et al.*, 1993). There is a strong intramolecular N—H···O hydrogen bond that stabilizes the molecular structure of the title compound. In the crystal structure, pairs of symmetry-related molecules are connected into centrosymmetric clusters *via* medium O—H···O and N—H···O hydrogen bonds forming twenty-six-membered rings with an *R*_6_^6^ (26) motif (Bernstein, *et al.*, 1995). The water molecule is surrounded by three organic groups through hydrogen bonds type O—H···O and N—H···O. The crystal packing (Fig. 2) is stabilized by these intermolecular hydrogen bonds and C—H···O interactions (Table 1) resulting in a three dimensional network.

### Experimental

An aqueous solution (10 ml) containing of 2,4-dinitro-phenylhydrazine (2 mmol) was added to pure acetic acid (10 ml). The obtained solution was stirred at 333 K for 30 min and then left to stand at room temperature. Yellow single crystals of the title compound were obtained after some days.

### Refinement

H atoms were treated as riding, with C—H = 0.93 and 0.96 A° for phenyl and methyl H-atoms, respectively. The H-atoms bonded to O and N were located from difference maps and were allowed to refine freely. The *U*_iso_(H) were allowed at 1.5*U*_eq_(C methyl) or 1.2*U*_eq_(C phenyl).

### Crystal data

**Table:**

C_8_H_8_N_4_O_5_·H_2_O	*F*(000) = 536
*M**_r_* = 258.20	*D*_x_ = 1.549 Mg m^−^^3^
Monoclinic, *P*2_1_/*c*	Ag *K*α radiation, λ = 0.56083 Å
Hall symbol: -P 2ybc	Cell parameters from 25 reflections
*a* = 7.702 (2) Å	θ = 9–11°
*b* = 7.057 (3) Å	µ = 0.08 mm^−^^1^
*c* = 21.550 (4) Å	*T* = 293 K
β = 109.044 (19)°	Prism, yellow
*V* = 1107.3 (6) Å^3^	0.5 × 0.4 × 0.3 mm
*Z* = 4	

### Data collection

**Table:**

Enraf–Nonius CAD-4 diffractometer	*R*_int_ = 0.064
Radiation source: fine-focus sealed tube	θ_max_ = 28.0°, θ_min_ = 2.2°
Graphite monochromator	*h* = −12→12
non–profiled ω scans	*k* = −11→2
7469 measured reflections	*l* = −36→14
5411 independent reflections	2 standard reflections every 120 min
2771 reflections with *I* > 2σ(*I*)	intensity decay: 1%

### Refinement

**Table:**

Refinement on *F*^2^	Secondary atom site location: difference Fourier map
Least-squares matrix: full	Hydrogen site location: inferred from neighbouring sites
*R*[*F*^2^ > 2σ(*F*^2^)] = 0.071	H atoms treated by a mixture of independent and constrained refinement
*wR*(*F*^2^) = 0.195	*w* = 1/[σ^2^(*F*_o_^2^) + (0.0627*P*)^2^ + 0.2081*P*] where *P* = (*F*_o_^2^ + 2*F*_c_^2^)/3
*S* = 0.98	(Δ/σ)_max_ = 0.004
5411 reflections	Δρ_max_ = 0.25 e Å^−^^3^
181 parameters	Δρ_min_ = −0.23 e Å^−^^3^
3 restraints	Extinction correction: *SHELXL97* (Sheldrick, 2008), Fc^*^=kFc[1+0.001xFc^2^λ^3^/sin(2θ)]^-1/4^
Primary atom site location: structure-invariant direct methods	Extinction coefficient: 0.027 (4)

### Special details

**Table:**

Geometry. All e.s.d.'s (except the e.s.d. in the dihedral angle between two l.s. planes) are estimated using the full covariance matrix. The cell e.s.d.'s are taken into account individually in the estimation of e.s.d.'s in distances, angles and torsion angles; correlations between e.s.d.'s in cell parameters are only used when they are defined by crystal symmetry. An approximate (isotropic) treatment of cell e.s.d.'s is used for estimating e.s.d.'s involving l.s. planes.
Refinement. Refinement of *F*^2^ against ALL reflections. The weighted *R*-factor *wR* and goodness of fit *S* are based on *F*^2^, conventional *R*-factors *R* are based on *F*, with *F* set to zero for negative *F*^2^. The threshold expression of *F*^2^ > σ(*F*^2^) is used only for calculating *R*-factors(gt) *etc*. and is not relevant to the choice of reflections for refinement. *R*-factors based on *F*^2^ are statistically about twice as large as those based on *F*, and *R*- factors based on ALL data will be even larger.

### Fractional atomic coordinates and isotropic or equivalent isotropic displacement parameters (Å^2^)

**Table:**

	*x*	*y*	*z*	*U*_iso_*/*U*_eq_	
OW	0.8341 (3)	0.6774 (3)	0.23696 (8)	0.0526 (5)	
H1W	0.784 (4)	0.583 (3)	0.2497 (13)	0.088 (11)*	
H2W	0.827 (4)	0.672 (5)	0.1972 (6)	0.091 (11)*	
H2N	0.894 (4)	0.171 (4)	0.1927 (14)	0.073 (9)*	
H1N	0.762 (3)	−0.050 (4)	0.2501 (11)	0.052 (7)*	
C1	0.6447 (3)	0.1614 (3)	0.12985 (10)	0.0332 (4)	
N2	0.7775 (3)	0.1390 (3)	0.18850 (8)	0.0416 (5)	
C2	0.6778 (3)	0.2383 (3)	0.07398 (10)	0.0347 (5)	
C4	0.3649 (3)	0.2020 (3)	0.00937 (10)	0.0387 (5)	
N3	0.8583 (3)	0.2980 (3)	0.07563 (10)	0.0467 (5)	
N4	0.2219 (3)	0.2185 (3)	−0.05403 (10)	0.0496 (5)	
C3	0.5376 (3)	0.2593 (3)	0.01484 (10)	0.0383 (5)	
H3	0.5615	0.3125	−0.0210	0.046*	
N1	0.7353 (3)	0.0698 (3)	0.24205 (9)	0.0410 (5)	
C6	0.4620 (3)	0.1034 (3)	0.12134 (10)	0.0385 (5)	
H6	0.4349	0.0515	0.1568	0.046*	
C5	0.3250 (3)	0.1218 (3)	0.06262 (11)	0.0422 (5)	
H5	0.2065	0.0815	0.0580	0.051*	
O5	0.6823 (3)	0.3600 (3)	0.27355 (9)	0.0607 (5)	
C7	0.6972 (3)	0.1892 (3)	0.28405 (10)	0.0388 (5)	
O4	0.2556 (3)	0.3098 (3)	−0.09698 (8)	0.0683 (6)	
O2	0.8788 (3)	0.3578 (3)	0.02501 (9)	0.0723 (6)	
O1	0.9859 (2)	0.2867 (3)	0.12714 (9)	0.0685 (6)	
C8	0.6708 (3)	0.1000 (4)	0.34342 (10)	0.0492 (6)	
H8A	0.7672	0.1403	0.3820	0.074*	
H8B	0.6741	−0.0355	0.3397	0.074*	
H8C	0.5542	0.1378	0.3465	0.074*	
O3	0.0748 (3)	0.1398 (3)	−0.06187 (10)	0.0722 (6)	

### Atomic displacement parameters (Å^2^)

**Table:**

	*U*^11^	*U*^22^	*U*^33^	*U*^12^	*U*^13^	*U*^23^
OW	0.0634 (11)	0.0483 (11)	0.0435 (10)	−0.0030 (9)	0.0139 (9)	0.0001 (8)
C1	0.0400 (11)	0.0272 (10)	0.0339 (10)	0.0000 (9)	0.0142 (9)	−0.0027 (9)
N2	0.0431 (10)	0.0511 (12)	0.0333 (9)	−0.0026 (10)	0.0161 (8)	0.0035 (9)
C2	0.0404 (11)	0.0309 (11)	0.0367 (10)	−0.0045 (9)	0.0179 (9)	−0.0028 (9)
C4	0.0432 (12)	0.0331 (11)	0.0358 (10)	0.0044 (10)	0.0074 (9)	−0.0024 (9)
N3	0.0510 (12)	0.0503 (12)	0.0443 (10)	−0.0083 (10)	0.0234 (10)	0.0013 (10)
N4	0.0566 (13)	0.0416 (11)	0.0436 (11)	0.0072 (10)	0.0068 (10)	−0.0066 (10)
C3	0.0543 (14)	0.0305 (11)	0.0339 (10)	−0.0001 (10)	0.0194 (10)	0.0016 (9)
N1	0.0534 (11)	0.0387 (11)	0.0342 (9)	0.0053 (9)	0.0188 (8)	0.0067 (9)
C6	0.0458 (12)	0.0372 (12)	0.0390 (11)	−0.0009 (10)	0.0226 (10)	0.0023 (10)
C5	0.0395 (12)	0.0386 (13)	0.0498 (13)	0.0002 (10)	0.0166 (10)	−0.0039 (10)
O5	0.0877 (13)	0.0382 (10)	0.0690 (12)	0.0093 (9)	0.0431 (11)	0.0063 (9)
C7	0.0399 (11)	0.0404 (13)	0.0378 (11)	0.0004 (10)	0.0152 (9)	0.0037 (10)
O4	0.0883 (14)	0.0697 (13)	0.0383 (9)	0.0014 (11)	0.0090 (9)	0.0087 (10)
O2	0.0695 (12)	0.1000 (16)	0.0573 (11)	−0.0166 (11)	0.0342 (10)	0.0137 (11)
O1	0.0465 (10)	0.1019 (16)	0.0551 (11)	−0.0231 (10)	0.0138 (9)	0.0105 (11)
C8	0.0548 (14)	0.0557 (16)	0.0397 (12)	0.0029 (12)	0.0188 (11)	0.0065 (11)
O3	0.0496 (10)	0.0774 (14)	0.0732 (13)	−0.0013 (11)	−0.0025 (9)	−0.0012 (11)

### Geometric parameters (Å, º)

**Table:**

OW—H1W	0.856 (10)	N4—O3	1.223 (3)
OW—H2W	0.841 (9)	N4—O4	1.224 (3)
C1—N2	1.351 (3)	C3—H3	0.9300
C1—C2	1.418 (3)	N1—C7	1.338 (3)
C1—C6	1.419 (3)	N1—H1N	0.88 (3)
N2—N1	1.386 (2)	C6—C5	1.364 (3)
N2—H2N	0.90 (3)	C6—H6	0.9300
C2—C3	1.384 (3)	C5—H5	0.9300
C2—N3	1.442 (3)	O5—C7	1.225 (3)
C4—C3	1.358 (3)	C7—C8	1.498 (3)
C4—C5	1.400 (3)	C8—H8A	0.9600
C4—N4	1.453 (3)	C8—H8B	0.9600
N3—O1	1.222 (2)	C8—H8C	0.9600
N3—O2	1.227 (2)		
			
H1W—OW—H2W	114 (2)	C4—C3—H3	120.1
N2—C1—C2	123.22 (18)	C2—C3—H3	120.1
N2—C1—C6	120.24 (18)	C7—N1—N2	120.3 (2)
C2—C1—C6	116.53 (18)	C7—N1—H1N	124.5 (16)
C1—N2—N1	120.55 (18)	N2—N1—H1N	113.5 (16)
C1—N2—H2N	119.4 (18)	C5—C6—C1	121.78 (19)
N1—N2—H2N	120.0 (18)	C5—C6—H6	119.1
C3—C2—C1	121.36 (18)	C1—C6—H6	119.1
C3—C2—N3	116.61 (18)	C6—C5—C4	119.25 (19)
C1—C2—N3	122.03 (18)	C6—C5—H5	120.4
C3—C4—C5	121.3 (2)	C4—C5—H5	120.4
C3—C4—N4	118.6 (2)	O5—C7—N1	121.5 (2)
C5—C4—N4	120.1 (2)	O5—C7—C8	122.7 (2)
O1—N3—O2	122.08 (19)	N1—C7—C8	115.7 (2)
O1—N3—C2	119.13 (17)	C7—C8—H8A	109.5
O2—N3—C2	118.79 (19)	C7—C8—H8B	109.5
O3—N4—O4	123.4 (2)	H8A—C8—H8B	109.5
O3—N4—C4	118.5 (2)	C7—C8—H8C	109.5
O4—N4—C4	118.1 (2)	H8A—C8—H8C	109.5
C4—C3—C2	119.74 (19)	H8B—C8—H8C	109.5

### Hydrogen-bond geometry (Å, º)

**Table:**

*D*—H···*A*	*D*—H	H···*A*	*D*···*A*	*D*—H···*A*
O*W*—H1*W*···O5	0.86 (1)	1.91 (1)	2.759 (3)	175 (3)
O*W*—H2*W*···O4^i^	0.84 (1)	2.05 (1)	2.868 (3)	165 (3)
N1—H1*N*···O*W*^ii^	0.88 (3)	2.04 (3)	2.883 (3)	160 (2)
N2—H2*N*···O1	0.90 (3)	1.96 (3)	2.607 (2)	128 (2)
N2—H2*N*···O*W*^iii^	0.90 (3)	2.15 (3)	2.910 (3)	142 (2)
C6—H6···O5^iv^	0.93	2.40	3.310 (3)	165

Symmetry codes: (i) −*x*+1, −*y*+1, −*z*; (ii) *x*, *y*−1, *z*; (iii) −*x*+2, *y*−1/2, −*z*+1/2; (iv) −*x*+1, *y*−1/2, −*z*+1/2.
